# Strategies to reduce low-value care – An applied behavior analysis using a single-case design

**DOI:** 10.3389/frhs.2023.1099538

**Published:** 2023-02-28

**Authors:** Sara Ingvarsson, Ingunn Sandaker, Per Nilsen, Henna Hasson, Hanna Augustsson, Ulrica von Thiele Schwarz

**Affiliations:** ^1^ Procome Research Group, Medical Management Centre, Department of Learning, Informatics, Management and Ethics, Karolinska Institutet, Stockholm, Sweden; ^2^ Department of Behavioral Science, Faculty of Health Sciences, Oslo Metropolitan University, Oslo, Norway; ^3^ Department of Health, Medical and Caring Sciences, Division of Public Health, Linköping University, Linköping, Sweden; ^4^ Unit for Implementation and Evaluation, Center for Epidemiology and Community Medicine (CES), Stockholm, Sweden; ^5^ School of Health, Care and Social Welfare, Mälardalen University, Västerås, Sweden

**Keywords:** low-value care, de-implementation, single-case design, primary care (MeSH), physicians, applied behavior analysis (ABA)

## Abstract

**Introduction:**

Implementation science has traditionally focused on the implementation of evidence-based practices, but the field has increasingly recognized the importance of addressing de-implementation (i.e., the process of reducing low-value care). Most studies on de-implementation strategies have used a combination of strategies without addressing factors that sustain the use of LVC and there is a lack of information about which strategies are most effective and what mechanisms of change might underlie these strategies. Applied behavior analysis is an approach that could be a potential method to gain insights into the mechanisms of de-implementation strategies to reduce LVC. Three research questions are addressed in this study: What contingencies (three-term contingencies or rule-governing behavior) related to the use of LVC can be found in a local context and what strategies can be developed based on an analysis of these contingencies?; Do these strategies change targeted behaviors?; How do the participants describe the strategies' contingencies and the feasibility of the applied behavior analysis approach?

**Materials and methods:**

In this study, we used applied behavior analysis to analyze contingencies that maintain behaviors related to a chosen LVC, the unnecessary use of x-rays for knee arthrosis within a primary care center. Based on this analysis, strategies were developed and evaluated using a single-case design and a qualitative analysis of interview data.

**Results:**

Two strategies were developed: a lecture and feedback meetings. The results from the single-case data were inconclusive but some of the findings may indicate a behavior change in the expected direction. Such a conclusion is supported by interview data showing that participants perceived an effect in response to both strategies.

**Conclusion:**

The findings illustrate how applied behavior analysis can be used to analyze contingencies related to the use of LVC and to design strategies for de-implementation. It also shows an effect of the targeted behaviors even though the quantitative results are inconclusive. The strategies used in this study could be further improved to target the contingencies better by structuring the feedback meetings better and including more precise feedback.

## 1. Introduction

Implementation science has traditionally focused on the implementation of evidence-based practices (1), but has lately also included the de-implementation of LVC (2). De-implementation is the process of reducing LVC (i.e., practices that lack scientific support for their efficacy or effectiveness and overuse of effective practices, such as patients that do not benefit and costs that exceed benefits) (3–6). The most common types of LVC are non-indicated antibiotics, potentially inappropriate medication for the elderly, unnecessary imaging, and unnecessary lab tests (7). One noticeable difference with de-implementation compared to implementation is that it often requires some health care professionals’ behaviors to be decreased (e.g., the use of a specific LVC practice) and some behaviors to be increased (e.g., the use of an alternative practice) (8). This implies that de-implementation needs to encompass strategies to decrease and increase behaviors.

Implementation science is accumulating knowledge about strategies. The current state-of-the-art is that strategies should match the local factors impacting behavior rather than expecting particular implementation strategies to always be superior to others (9). With regard to de-implementation, numerous local factors have been found to influence the use of LVC, including care processes, financial incentives, and perceived pressure from patients, other professionals, or the system (7, 10–12). However, there is insufficient knowledge about which factors might be relevant for choosing effective strategies. Knowledge is also required to determine which mechanisms are needed to target a factor (13, 14). Mechanisms are the processes or events responsible for the changes produced by a strategy (15). In other words, mechanisms explain how or why a strategy works by providing a specific description on how the factors influencing behaviors are altered in a given context (14). Thus far, only a few studies have explored the mechanisms behind strategies for implementation and de-implementation (14). Understanding the local factors influencing the use of LVC and mechanisms of possible strategies could help to design strategies that focus both on increasing and decreasing the behaviors influencing the use of LVC.

Behavior change theories, such as the theory of planned behavior and operant learning theory, have been proposed as suitable methods for understanding mechanisms of strategies (16). Specifically, operant learning theory has been suggested to be related to de-implementation because it distinguishes between processes to increase and decrease behaviors (17). It is commonly referred to as applied behavior analysis, which focuses on how behaviors are established, maintained, and extinguished in response to their environment (18, 19). In applied behavior analysis, mechanisms are represented as so-called contingencies, including which contingencies maintain current behaviors and how these contingencies can be changed through different behavior change strategies. Contingencies can either be related to antecedents and consequences in the environment (the three-term contingency) or to rule-governing behaviors. Applied behavior analysis could be a valuable addition to further researchers' understanding of factors in the environment that maintain behaviors related to the use of LVC and how de-implementation strategies can be designed to reduce the use of LVC.

This study demonstrates how applied behavior analysis can be used to understand contingencies related to the use of LVC and how de-implementation strategies can be developed by arranging alternative contingencies. We will also present how a commonly used evaluation method within applied behavior analysis called single-case design can be used.

Three research questions were addressed:

(1) What contingencies related to the use of LVC can be found in a local context and what strategies can be developed based on an analysis of these contingencies?
(2) Do these strategies change targeted behaviors?
(3) How do the participants describe how the strategies influenced contingencies and the feasibility of the applied behavior analysis approach?

## 2. Materials and methods

In this study, we used applied behavior analysis to develop de-implementation strategies for LVC. The strategies were evaluated using a single-case design for an analysis of quantitative data to address research question 2 and a qualitative design for an analysis of interview data to address research questions 2 and 3.

The methods section describes the setting and recruitment and presents the key principles and procedures of the applied behavior analysis. This is followed by a description of the single-case design methodology and the qualitative analysis methods.

### 2.1. Setting and recruitment

The study was set within a primary care center in Stockholm, Sweden. The Swedish health care system is tax funded and consists of 21 regions throughout Sweden, with Stockholm having the largest population (2.5 million). Each region is responsible for the provision of care, including primary care, of its citizens (20).

This center was recruited from managers in primary care centers that previously participated in an explanatory interview study that aimed to describe management strategies related to the use of LVC (21). All 12 managers that participated in the previous study were invited to this study. Three managers expressed initial interest and after an information meeting, one agreed to participate. The participating center is publicly owned, has approximately 12,500 listed patients, and 12–13 employed physicians, which is a slightly above average for a primary care center in Region Stockholm. During this study, a total of 23 different physicians worked at the center, with 12–13 working per month.

### 2.2. Key principles of applied behavior analysis

Applied behavior analysis is a practical approach that has been used to achieve behavior change in various settings, including health care organizations (22). It has previously been used to increase staff attendance (23), improve compliance with routines (24–26), and increase emergency department efficiency (27). It has also been used to understand the mechanisms underlying management strategies to de-implementation (21). However, its potential contribution to implementation science has not been fully realized yet.

One of the key principles within applied behavior analysis is the three-term contingency (28). This involves the assumption that behaviors are maintained, changed, or extinguished through a combination of behavior antecedents (an event that precedes the behavior) and behavior consequences (an event that follows the behavior) (29, 30) (see Table 1 for key principles and concepts). Known factors that influence the use of LVC, such as expressed expectations from a patient, can be both an antecedent (the expressed expectation of receiving the LVC) and a consequence (the expressed thanks or relief from the patient after receiving the LVC). To design a strategy to influence the use of LVC, these contingencies need to be changed to support behavior change.

**Table 1:** Key principles and concepts within applied behavior analysis.

Key principle	Concepts	Description
Three-term contingency	Antecedent	An event that precedes and signals an expected behavior and the consequences that will follow.
	Consequences	An event that comes after the behavior that maintain, change, or extinguish behaviors.
Rule-governing	Rule	An instruction that states the expected behavior and the expected consequences for performing the behavior.

Another key principle is rule-governed behaviors (28), which are behaviors that are learned without having experienced the real-life consequences (31). Rules usually state the expected behavior and the consequences that will follow. Many of our behaviors are learned through rules (32). This is necessary when the process of trial and error is too time-consuming or could have a severe negative impact. For instance, in medical education, it is not acceptable to use trial and error to learn advanced medical procedures, but instructions (rules) can speed up learning. This makes rules a powerful tool for influencing behaviors.

Behaviors learned through rule-governing tend to be more inflexible and less influenced by antecedents and consequences. If a behavior needs to be robust in an environment where there are antecedents and consequences that encourage less suitable behaviors, using rule-governing can be beneficial. In contrast, when behaviors need to be flexible in a changing environment, rule-governing can instead cause problems. The factors influencing the use of LVC, such as uncertainty or disagreement about what is considered LVC, could be related to a lack of a clear rule that states what practices to avoid or the presence of a competing rule suggesting that the practice should be used.

### 2.3. The applied behavior analysis procedure

To develop strategies based on applied behavior analysis, we applied a six-step process (29) adapted for de-implementation (see Table 2). All of these steps are preferably performed together with the managers and employees to combine their knowledge about the local context with the researchers' expertise in behavioral analysis. All six steps were followed in this study. In addition to the six steps described in the literature, we also explored how the participating physicians described how the strategies influenced contingencies and the feasibility of the applied behavior analysis approach.

**Table 2:** Process for developing and evaluating strategies based on applied behavior analysis (adapted for de-implementation).

1. Specify which LVC to de-implement. 2. Identify specific behavior changes related to the use of that LVC. 3. Develop an accurate and reliable means of measuring key results and/or behaviors. 4. Conduct an analysis of the contingencies influencing behaviors related to the chosen results. 5. Develop and implement strategies targeting those contingencies. 6. Track and evaluate the effects of the strategies.

#### 2.3.1. Step 1. Specify which LVC to de-implement

X-rays for knee arthrosis was chosen as the target LVC based on a participatory process involving physicians and the manager at the center. The project was presented at a physician meeting (May 2021) and different examples of LVC that might be relevant based on the literature and local relevance were discussed. The manager made the final decision on which LVC practice to de-implement. The choice was justified based on a new guideline advising against overuse of this particular examination (33) and existing data indicating that the center had a higher use of the practice compared to other centers in the region.

Arthrosis causes degeneration of cartilage in the knee capsule that over time can become gradually more painful, making it difficult for patients to move naturally. Updated guidelines from the National Board of Health and Welfare in Sweden (33) were published in January 2021, which recommended that patients with suspected knee arthrosis be provided a diagnosis based on medical history, clinical symptoms, and a physical examination. The guidelines do not recommend ordering an x-ray unless the patient is referred to an orthopedic specialist for surgical treatment. The recommended treatment for knee arthrosis is physical therapy, weight loss (if relevant), pain medication, and physical aids. Surgery is the last step, and only then may an x-ray be necessary. There are several reasons why an x-ray is considered LVC for knee arthrosis: It exposes the patients to unnecessary radiation, it is costly, and it delays the diagnosis and, by extension, the treatment for the patients. Lastly, in the early stages of arthrosis, it is not always possible to verify a patient's condition through an x-ray examination (33).

#### 2.3.2. Step 2. Identify behaviors related to the unnecessary use of x-rays

Three behaviors related to the unnecessary use of x-rays for knee arthrosis were identified as targets for change: (1) a decrease of referring patients to x-ray examination when the examination was not warranted; (2) an increase of diagnosing patients with arthrosis without using an x-ray (by clinical assessment); and (3) a decrease of diagnosing patients with general knee pain while waiting for the results of the unnecessary x-ray. Identification of behavior changes were performed by the first author of this study, who is trained in applied behavior analysis and the manager at the center.

#### 2.3.3. Step 3. Develop an accurate and reliable means of measuring key results and/or behaviors

X-ray use and diagnoses of arthrosis and general knee pain were measured with data from the centers administrative registers and the quality assurance system. The monthly number of x-rays ordered at the primary care center was collected from central administrative register by their administrative staff and the use of the two diagnoses per month was collected from the local quality assurance system by the medically responsible physician at the primary care center. All data was on center level; it was not possible to extract data on an individual level.

#### 2.3.4. Step 4. Conduct an analysis of the contingencies influencing behaviors related to the chosen results (research question 1)

Contingencies relevant to the general use of LVC at the center were discussed at a meeting with all physicians at the center (May 2021). Two of the authors facilitated the discussion (SI and HH). Afterwards, SI and the manager further investigated the chosen LVC (i.e., unnecessary use of x-ray for knee arthrosis). The discussion with the physicians and the managers did not use technical jargon or terms from applied behavior analysis but rather featured questions such as what they believed might influence unnecessary use of x-rays. The answers were then categorized using the three-term contingency and rule-governed behavior.

#### 2.3.5. Step 5. Develop and implement strategies targeting the identified contingencies (research question 1)

At the meeting with physicians, possible strategies to reduce LVC in general were discussed. Strategies were developed based on a combination of the physicians' general suggestions and a specific discussion with the manager related to the chosen LVC practice. The suggested strategies were evaluated by the researchers based on their expected impact on the identified contingencies influencing unnecessary use of x-rays. As a result, the strategies were classified as either influencing rule-governing behavior or three-term contingencies related to unnecessary use of x-rays. In addition, this study's choice of strategies was also guided by how feasible the strategies were to implement without using too many of the center's resources.

#### 2.3.6. Step 6. Track and evaluate the effects of the chosen strategies (research question 2)

To evaluate whether the chosen strategies changed the target behaviors, we assessed three outcomes: (1) the number of x-rays ordered (expected to decrease); (2) the number of patients diagnosed with arthrosis (expected to increase); and (3) the number of patients with a less specific diagnosis of knee pain (expected to decrease). All outcomes were directly linked to the targeted behaviors as ordering an x-ray (behavior) is directly translatable to number of x-rays ordered. Only collective data on center level (i.e., not at the individual physician level) were available. However, this outcome was deemed relevant since the strategies were developed to target everyone working at the center.

##### 2.3.6.1. Single-case design

The effects were tracked using a single-case design, which is common in applied behavior analysis because it aligns with a perspective of science that emphasizes understanding “the black box” of change by closely monitoring the behavior of interest and how it changes following the adjustment of factors believed to influence the behavior (i.e., by applying strategies that change the three-term contingency or rule-governed behavior). Rather than evaluating changes in outcomes for groups of units (i.e., individuals, workplaces) before and after an intervention, a single-case design involves studying behavior change for each unit separately by using several data points over time and by distinguishing between a baseline phase and one or several intervention phases (34). To distinguish between the effects of different strategies, each strategy can be tracked through several data points to offer them time to influence behavior before another strategy is presented. The single-case data will be presented according to the Single-Case Reporting Guideline in Behavioral Interventions (35).

Following a single-case design, the data were collected each month for a period of 15 months (from June 2021 to August 2022) during four phases for all three outcomes.

Phase A: Baseline (no strategy introduced); six months before the introduction of the first strategy (i.e., June to November 2021).
Phase B: Three months after the introduction of the first strategy (i.e., December 2021 to February 2022).
Phase C: Three months after the introduction of the second strategy (i.e., March to May 2022).
Phase D: Follow-up (i.e., June to August 2022).

##### 2.3.6.2. Analysis of single-case data

To analyze the single-case design data, a graphic presentation of the data was visually analyzed following the standards for single-case design (28, 36) (see Table 3).

**Table 3:** Standards for single-case design: four steps and six features for analyzing single-case design data. .

**Steps:** Step 1. Documenting a predictable and stable baseline Step 2. Examining data within each phase to determine the pattern with each phase Step 3. Comparing visual data between each phase to interpret if the implemented strategies influenced the data Step 4. Integrating the information from all phases to evaluate if there is any demonstration of an effect **Features:** (a) level (b) trend (c) variability (d) immediacy of the effect (e) overlap in data between phases (f) consistency of data patterns across similar phases

A predictable and stable baseline involves a consistent pattern in level or trend. A consistent pattern in level means that all or most data points are on a similar level and a trend could be stable, increasing, or decreasing. Examining data within each phase to determine the pattern also involves finding a consistent pattern in level or trend. Comparisons between phases means looking at similarities or differences in level, trend, or variability. If differences are found, the immediacy of the effects involves if the change happens at the first data point for the new phase or gradually over time during the phase. Overlap in data between phases involves an analysis of how many of the data points in the phases overlap with data points of the comparing phase. Consistency of data patterns across similar phases involves analyzing if similar phases, such as baseline phases, show a similar pattern or if intervention phases are similar. This feature is difficult to apply to this study because there were two different strategies and follow-up is not likely to function as a return to baseline.

In addition to visual analysis of the data, the mean and standard deviation were calculated for each phase. Differences between the phases were evaluated using Cohen's *d* for effect size, and the overlap between phases was evaluated using the Nonoverlap of All Pairs (NAP) (37).

##### 2.3.6.3. Interviews

In addition to exploring how the strategies changed target behaviors using a single-case design, we also conducted individual interviews with the participants to capture their perception of the effect of the chosen strategies. The interviews were held after the strategies were implemented (May and June 2022). All physicians in the center were invited to participate in the interviews (*n* = 12), and four agreed to participate. In addition, all physicians who participated provided written consent. A semi-structured interview guide was used. The questions focused on their views on the specific LVC, how they perceived the strategies, and the usefulness/feasibility of the design and evaluation process. Questions on the strategies included aspects they felt did not work well, how the strategies could be improved, and if the strategies were perceived as feasible to use for the de-implementation of the other LVC.

##### 2.3.6.4. Analysis of the interviews

The interviews were recorded and transcribed verbatim. Data from the interviews were analyzed using conventional content analysis according to Graneheim and Lundman (38) using NVivo software. The transcribed interviews were first read through several times to obtain a general view of the material. The first author then inductively coded, using line-by-line coding. The codes were then grouped into preliminary categories. During this time, memos were written to capture general ideas related to the interpretation of the codes. These ideas were then tested in the data, and the first author revised the categories. Representative quotes were selected to illustrate the categories. All authors reviewed the final categories and quotes.

#### 2.3.7. Analyzing how the participants describe the contingencies of the strategies and the feasibility of the approach – research question 3

Data from the interviews related to the contingencies and the feasibility were analyzed separately. All answers were first coded inductively using content analysis. Answers related to contingencies were then coded deductively using the concepts from applied behavioral analysis three-term contingency and rule-governed behavior. This was done both for the contingencies that participants had pointed out as influencing their use of the chosen LVC and the lack thereof. Finally, the answers related to the feasibility of the design process and evaluation method were coded inductively using content analysis.

## 3. Results

The results section is divided into three subsections, each responding to a different research question.

### 3.1. What contingencies related to the use of LVC can be found in a local context and what strategies can be developed based on an analysis of these contingencies (RQ1)?

**Figure 1:**
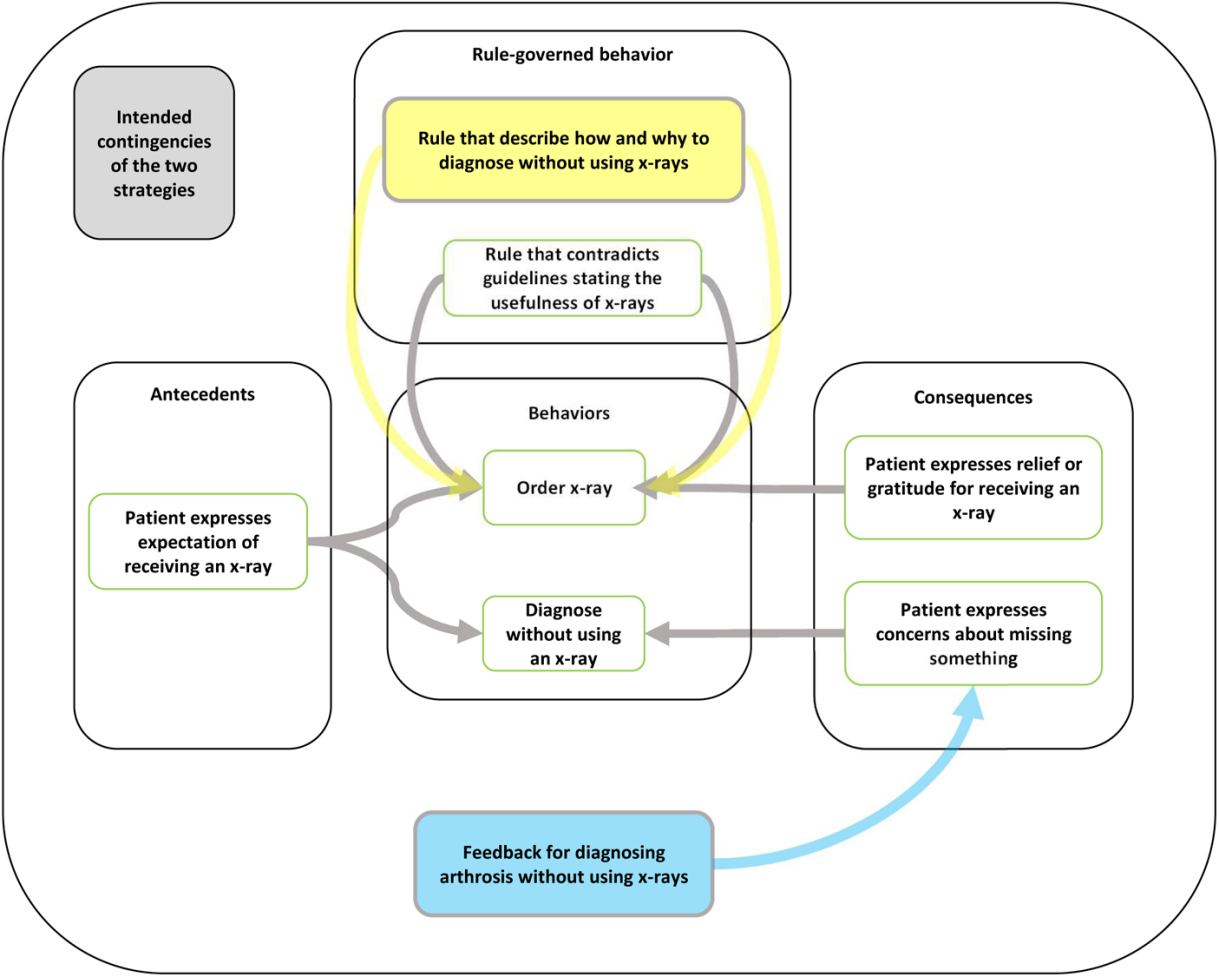
Connecting strategies to the analysis of contingencies specific to the unnecessary use of x-rays for arthrosis using applied behavior analysis. The yellow box shows how the lecture introduced a new rule to govern behaviors related to diagnosing arthrosis without using an x-ray. The blue box shows how the feedback meetings would add a new consequence to encourage the participants to diagnose arthrosis without using an x-ray.

Based on the information received through the meetings with the physicians and the manager of the center, an applied behavior analysis was conducted to identify antecedents and consequences and rules governing LVC behavior (Figure 1). The analysis indicated that the most important reasons for using x-rays (i.e., the contingencies) were for cases when patients expressed their expectation to receive an x-ray to diagnose their symptoms (an antecedent to order an x-ray) and when they reacted in the form of expressed relief or gratitude for receiving an x-ray when the physicians ordered one (a consequence reinforcing the behavior ordering an x-ray). A rule-governing behaviors related to ordering x-rays was that if you order an x-ray (behavior), the patient can be better diagnosed (expected consequence of the behavior).

Based on the contingencies, two strategies were developed: a lecture and feedback meetings. The first strategy, the lecture, aimed to introduce a competing rule-governing the chosen behaviors, specifying why they should not order x-rays for arthrosis unless for referral to an orthopedic surgeon, how to diagnose arthrosis without ordering an x-ray, and what warning signs to be aware of when diagnosing arthrosis to avoid missing an alternative diagnosis. The new rules would be: do not order an x-ray unless the patient is eligible for knee surgery, and: if you diagnose knee arthrosis without using an x-ray (behavior) the patient will faster receive the correct treatment (expected consequence of the behavior). The lecture was held by a physiotherapist at a rehab center with which the primary care was already collaborating. The lecture was planned in collaboration with the manager and the medically responsible physician, and during the meeting they expressed their support for following the new guideline. The physiotherapist presented verbally and through a PowerPoint presentation the national guidelines for diagnosing and treating arthrosis state that an x-ray is not recommended. The lecture included a hierarchy of treatment options depending on the severity of the symptoms, a description about why one should not order unnecessary x-rays for arthrosis, how to diagnose arthrosis without using an x-ray examination, and why one does not need to use the general knee-pain diagnosis. Compared to the previously published guideline with the span of 80 pages of single-spaced lines, the instructions were brief and formatted as bullet points to clarify which specific behaviors were according to the guideline in an accessible way. The instruction also included so-called red flags and a checklist for symptoms to be vigilant about in order to avoid missing a more serious diagnosis, still without having to order an x-ray. The lecture was delivered face to face in group format, attended by all physicians at the center. The entire lecture was 45 min, of which the presentation was around 20 min, and the remaining 25 min were used to give the participants the opportunity to ask questions and discuss the information.

The second strategy, feedback meetings, aimed at influencing the three-term contingencies related to the chosen behaviors by adding a consequence related to diagnosing arthrosis without using an x-ray. The new three-term contingency would then be: patient expresses expectations on receiving an x-ray (antecedent), diagnose arthrosis without using an x-ray (behavior) to receive feedback and support from colleagues and the medically responsible physician (consequence). A total of three meetings were held monthly and were hosted by the medically responsible physician whose responsibilities included quality of care. During the meetings, one of the researchers (SI) presented data on how the center was performing in three areas: how many knee x-rays had been ordered, how many patients had been diagnosed with arthrosis, and how many patients had been diagnosed with general knee pain. The meetings aimed at lessening the effects of the pre-existing contingencies related to using unnecessary x-rays for diagnosing arthrosis by increasing antecedents and consequences to diagnosing arthrosis without using unnecessary x-rays. Antecedents included discussions on what clinical signals should function as antecedents for ordering or not ordering an x-ray and consequences in terms of receiving support from colleagues and the medically responsible physician for not diagnosing patients with arthrosis without using an x-ray.

Examples of discussions held at both the lecture and the feedback meetings were (1) how to communicate with patients who strongly request an x-ray; (2) how to feel secure that the patients' symptoms were not related to a more severe diagnosis (e.g., cancer); (3) lack of correlation between visible arthrosis on an x-ray and severity of the symptoms for the patient; and (4) problems with convincing patients that physiotherapy would be helpful for their symptoms.

All physicians at the center were invited to participate in the lecture and the feedback meetings. Ten participated in the lecture, six in the first feedback-meeting, five in the second, and four in the third. The number of participants per meeting depended on how many physicians were at the center on the day of the meeting.

### 3.2. Do these strategies change targeted behaviors (RQ2)?

The findings regarding the use of x-rays, arthrosis diagnosis, and general knee-pain diagnosis are presented using visual and statistical examination of the data.

#### 3.2.1. Use of x-rays

**Figure 2:**
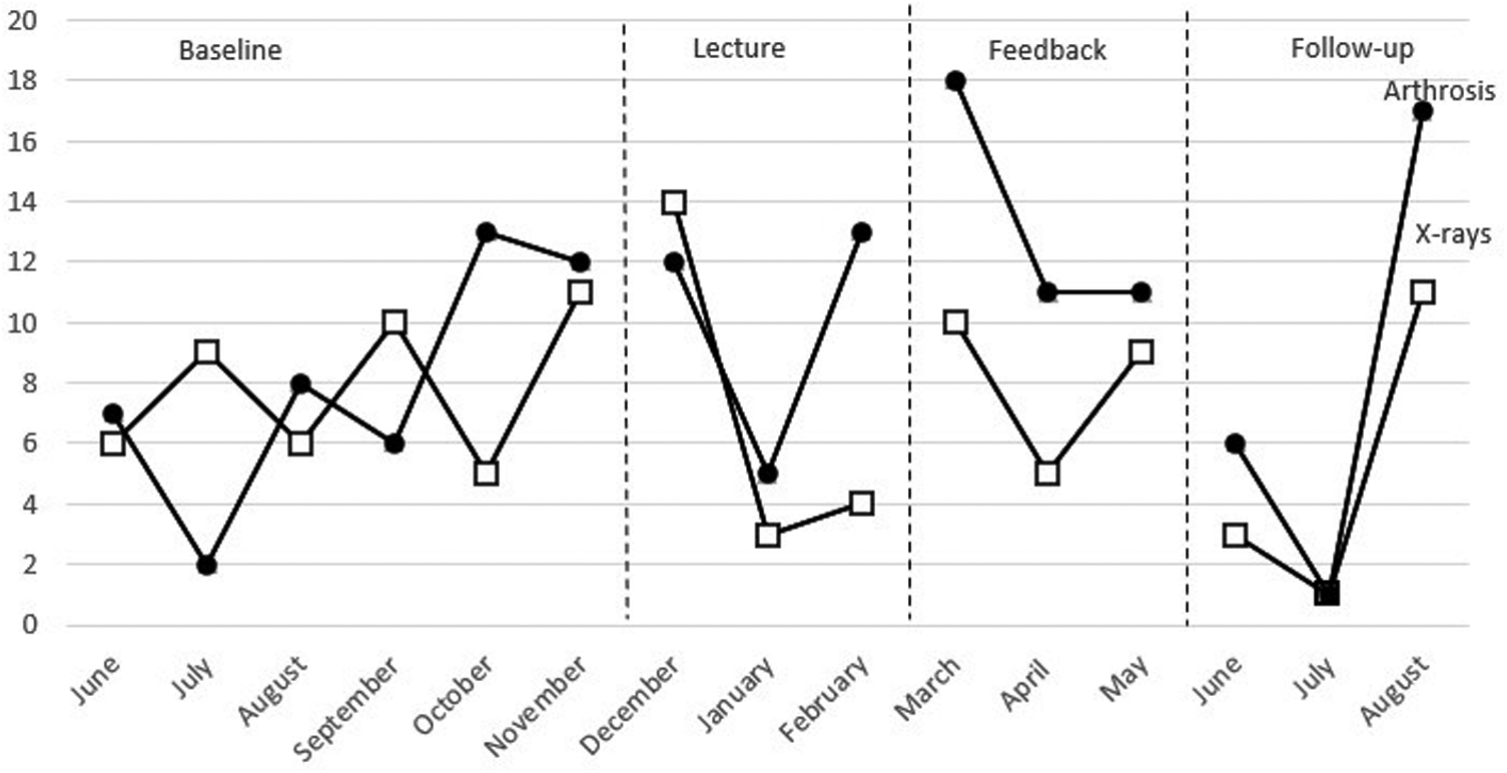
Combined data from number of x-rays ordered and number of patients receiving arthrosis diagnosis.

During the baseline phase, the number of x-rays ordered per month varied but remained relatively stable around a mean value of 7.8 x-rays ordered per month showing a predictable and stable baseline (step 1) (Figure 2). Additional visual presentation of the single-case design data can be found in APPENDIX 1 (Supplementary Material). When examining the data within each of the four phases to determine the pattern of each phase (step 2), the baseline phase showed an increasing trend and a low variation. During the lecture phase, there was a decreasing trend and an increased variation with a mean of 7.0 x-rays per month, varying from 3 to 14 x-rays ordered per month. The feedback phase had a decreasing trend and smaller variation with a mean of 8.0 x-rays per month. The fourth phase, follow-up, had an increasing trendline and a low variation.

When comparing the visual data between each phase to interpret if the strategies influenced the data (step 3), there was a difference in level, trend, and variability between the baseline and the lecture phase but no clear immediate effect. The first data point in the lecture phase was higher than all points in the baseline phase, and the two following data points were lower than all data points in the baseline. During the feedback phase, there was a difference in trend, but not in level and variability, compared to the baseline phase, and there was a difference in level and variability compared to the lecture phase. The difference in level was immediate compared to the lecture phase. All data points in the feedback phase overlapped with the data points in the baseline phase. The follow-up phase had a lower level than all other phases and an increasing trend similar to the baseline but a larger variability. The first two data points in the follow-up phase overlapped with none of the other phases, whereas the third data point overlapped with one data point per phase. There was no consistency of data patterns across the different phases (step 4).

The statistic measure NAP between phases shows there were a large number of nonoverlapping pairs in the lecture phase compared to the baseline (Table 4). The NAP was lower when comparing the baseline to the feedback phase and higher when comparing the baseline to the follow-up. None of the NAP for the baseline compared to the other phases was significant. The effect size calculations indicate no large effects.

**Table 4:** Number of patients referred to an x-ray: the mean value and standard deviation for the four phases and effect size using Cohen's *d* and nonoverlap of All pairs (NAP).

	Baseline	Lecture	Feedback	Follow-up
Mean	7.8	7.0	8.0	5.0
Standard deviation	2.5	6.1	2.6	5.3
Effect size compared to baseline Cohen's *d*		0.18	0.7	0.7
Effect size compared to baseline NAP		67	53	69
*p*-value NAP		0.26	0.5	0.22

#### 3.2.2. Arthrosis

**Figure 3:**
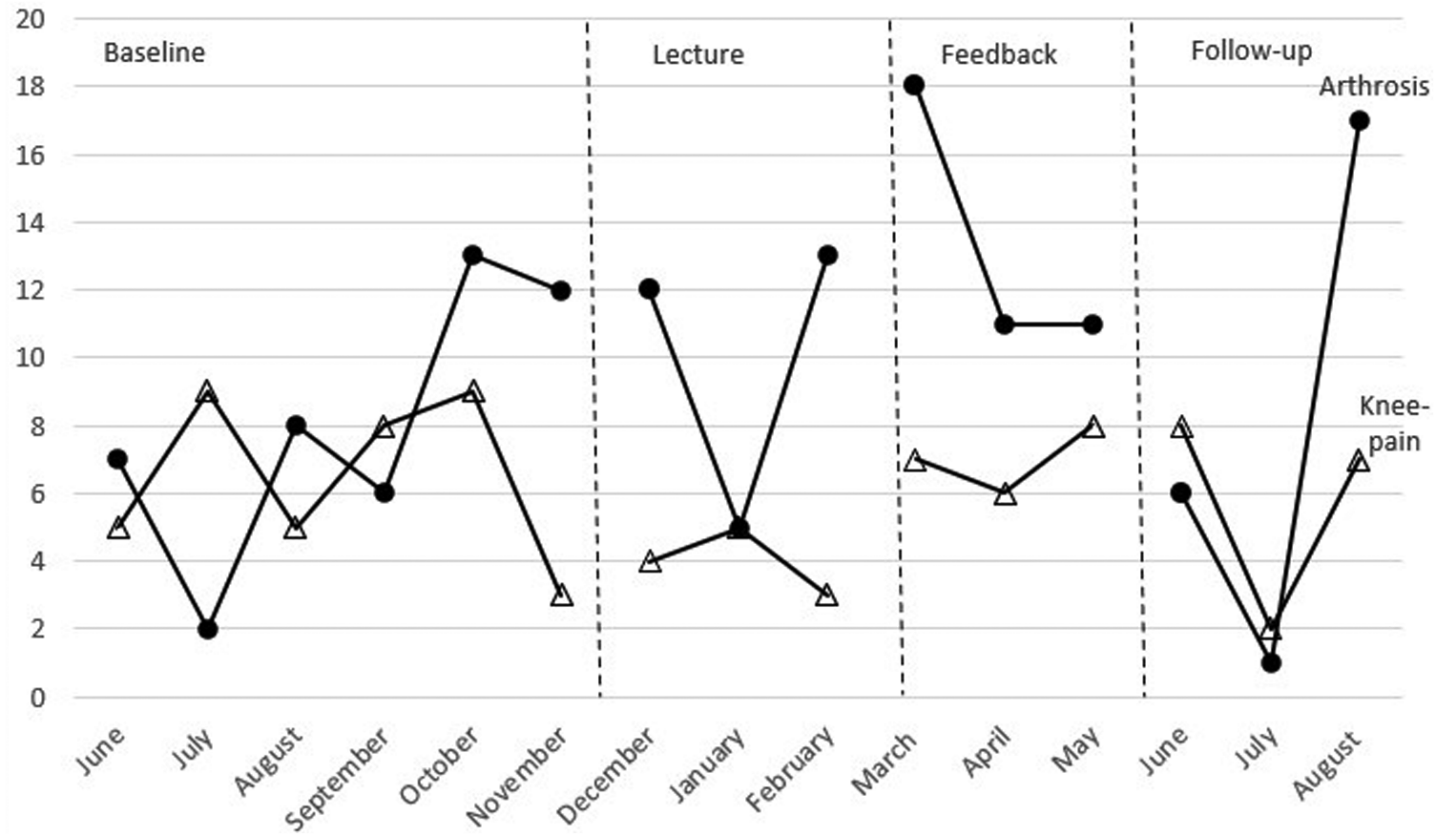
Combined data from the number of patients receiving arthrosis diagnosis and knee-pain diagnosis for the four phases.

Data for the number of patients receiving arthrosis diagnosis can be seen in Figure 2 (compared with x-rays) and Figure 3 (compared with the number of patients receiving the diagnosis of general knee pain). Additional visual presentation of the single-case design data can be found in APPENDIX 1 (Supplementary Material). During the baseline phase, the number of patients receiving arthrosis diagnosis showed a large variation with a mean of 8.0 per month, demonstrating that the baseline phase was not predictable and stable (step 1). When examining the data within each phase to determine the pattern of every phase (step 2), the baseline phase showed an increasing trend and a large variation. The lecture phase shows an increasing trend and a large variation. The mean number of patients receiving the diagnosis was 10.0 per month. The feedback phase had a decreasing trend, and there was a small variation. The mean value was 13.3. During follow-up, the trend was increasing, and there was a large variation. The mean was 8.0.

Comparing visual data between each phase to interpret if the strategies influenced the data (step 3), there was a difference in level and variability between the baseline and the lecture phase. There was no immediate effect between the two phases. The feedback had a higher level than both the baseline and the lecture phase, a variability similar to the lecture phase, and a different trend (decreasing) compared to both the baseline and the lecture phase. There was an immediate effect between the lecture phase and the feedback phase. The follow-up phase had a level similar to the baseline, a larger variability than all other phases, and an increasing trend like the baseline and the lecture phase. There was an immediate effect between the feedback phase and the follow-up phase. There was no consistency of data patterns across the different phases (step 4).

NAP indicates there were a small number of nonoverlapping pairs in the lecture phase compared to the baseline (Table 5). The NAP was higher when comparing the baseline to the feedback phase and lower when comparing the baseline to the follow-up. None of the NAP had a significant *p*-value. Only the feedback phase compared to the baseline had a large effect size.

**Table 5:** Number of patients receiving arthrosis diagnosis: the mean value and standard deviation for the four phases and effect size using Cohen's *d* and nonoverlap of All pairs (NAP).

	Baseline	Lecture	Feedback	Follow-up
Mean	8.0	10.0	13.3	8.0
Standard deviation	4.0	6.0	4.0	8.2
Effect size compared to baseline Cohen's *d*		0.5	1.3	0.0
Effect size compared to baseline NAP		61	78	42
*p*-value NAP		0.35	0.12	0.7

#### 3.2.3. General knee pain

Data for the number of patients receiving arthrosis diagnosis can be seen in Figure 3 (compared to the number of patients receiving arthrosis diagnosis). Additional visual presentation of the single-case design data can be found in APPENDIX 1 (Supplementary Material). During the baseline, there was a large variation suggesting that the baseline phase was not predictable and stable (step 1). When examining the data within each phase to determine the pattern of each phase (step 2), the baseline phase had a large variation and a decreasing trend. The mean value of patients received the diagnosis of 6.7 per month. The lecture phase has a decreasing trend and a small variation. The mean number of patients receiving the diagnosis was 4.0 per month. The feedback phase had an increasing trend and a small variation. The mean value was 7.0. The follow-up phase had a decreasing trendline and a small variation and had the mean value of 5.7.

Comparing visual data between each phase to interpret if the strategies influenced the data (step 3), there was a difference in level and variability between the baseline phase and the lecture phase. There was no immediate effect. The feedback phase had a similar level as the baseline phase but a decreasing trend compared to all other phases. There was an immediate effect between the lecture phase and the feedback phase. The follow-up phase had a trend similar to all phases except for the feedback phase, a lower level than the baseline and the feedback phase, and the same variability as the baseline. There was no immediate effect between the feedback phase and the follow-up phase. There was no consistency of data patterns across the different phases (step 4).

NAP indicates there were a high number of nonoverlapping pairs in the lecture phase compared to the baseline (Table 6). The NAP was lower when comparing baseline to the feedback phase and higher when comparing baseline to follow-up. None of the NAP has a significant *p*-value. Only the lecture phase compared to the baseline had a large effect size.

**Table 6:** Number of patients receiving the general knee-pain diagnosis: the mean value and standard deviation for the four phases and effect size using Cohen's *d* and nonoverlap of all pairs (NAP).

	Baseline	Lecture	Feedback	Follow-up
Mean	6.5	4.0	7.0	5.7
Standard deviation	2.5	1.0	1.0	3.2
Effect size compared to baseline Cohen's *d*		1.3	0.26	0.29
Effect size compared to baseline using NAP		81	47	64
*p*-value NAP		0.09	0.6	0.3

#### 3.2.4. Combining x-rays and arthrosis data

Inspecting the combined data from x-rays and arthrosis revealed that the number of x-rays ordered exceeded the number of patients receiving arthrosis diagnosis on two occasions during the baseline and at the first data point during the lecture phase, but not after that, suggesting that more patients received unnecessary x-rays before the lecture.

#### 3.2.5. Combining arthrosis and knee pain data

The combined data from arthrosis and knee-pain diagnosis showed a similar pattern, with more data points in the baseline where the number of patients diagnosed with knee pain exceeded the number of patients receiving arthrosis diagnosis, suggesting that more patients received an unnecessary knee-pain diagnosis, whereas the opposite pattern appeared in the strategy and follow-up phases (Figure 3).

### 3.3. Qualitative data: do the strategies change targeted behaviors?

All participants described that the strategies had influenced their use of LVC. These effects can be grouped into four categories: (1) noticing that their own use of x-rays had been reduced; (2) talking more to patients about the lack of benefit from using x-rays; (3) improving their way of diagnosing arthrosis without using x-rays; and (4) being unsure of how to interpret the effect.

The category Noticing that their own use of x-rays had been reduced were related to their subjective perception of how many x-rays they had ordered since the implementation of the first strategy. Some described that they had ceased using x-rays except for when referring to a specialist in orthopedic surgery, whereas others said they were more aware of when they ordered an x-ray that was unnecessary and that they were more selective when doing so.

“I don’t believe I have ordered any since the lecture—well, yes, some—but those were related to a referral to an orthopedic surgeon” (IP2).

The category “Talking more to patients about the lack of benefit from using x-rays” summarized physicians' descriptions of how they talked more to patients about the lack of benefit from using x-rays after the strategies had been implemented. They described using phrases that they picked up from the lecture and from discussions with their colleagues during the feedback meetings in their conversations with patients.

“I believe that it has influenced my use of x-rays, by how I talk to the patients—that an x-ray is not needed until it is time for surgery” (IP3).

The category “Improving their way of diagnosing patients without using an x-ray” included both how to diagnose patients with arthrosis without using an x-ray and the importance of doing so. Physicians described new insights about x-rays potentially leading to missed or delayed diagnoses because the symptoms of arthrosis are not visual on an x-ray until late in the development of the disease.

“What resonated with me especially was that there is a risk that we miss diagnosing patients with arthrosis if we wait for an x-ray, and if that doesn’t show anything, we do not trust our own assessment of the patient's symptoms” (IP4).

The category “Being unsure of how to interpret the effect” included statements about difficulties in interpreting the feedback received during the feedback sessions and the confidence in their own perception of change. All participants perceived that there had been an effect, but some were not sure about how and to what extent the strategies had led to effects.

“I like to think that it has influenced our way of thinking, but I am not sure” (IP1).

### 3.4. Qualitative data – how do the participants describe the contingencies of the strategies and the feasibility of the applied behavior analysis approach (RQ3)?

#### 3.4.1. Contingencies

**Figure 4:**
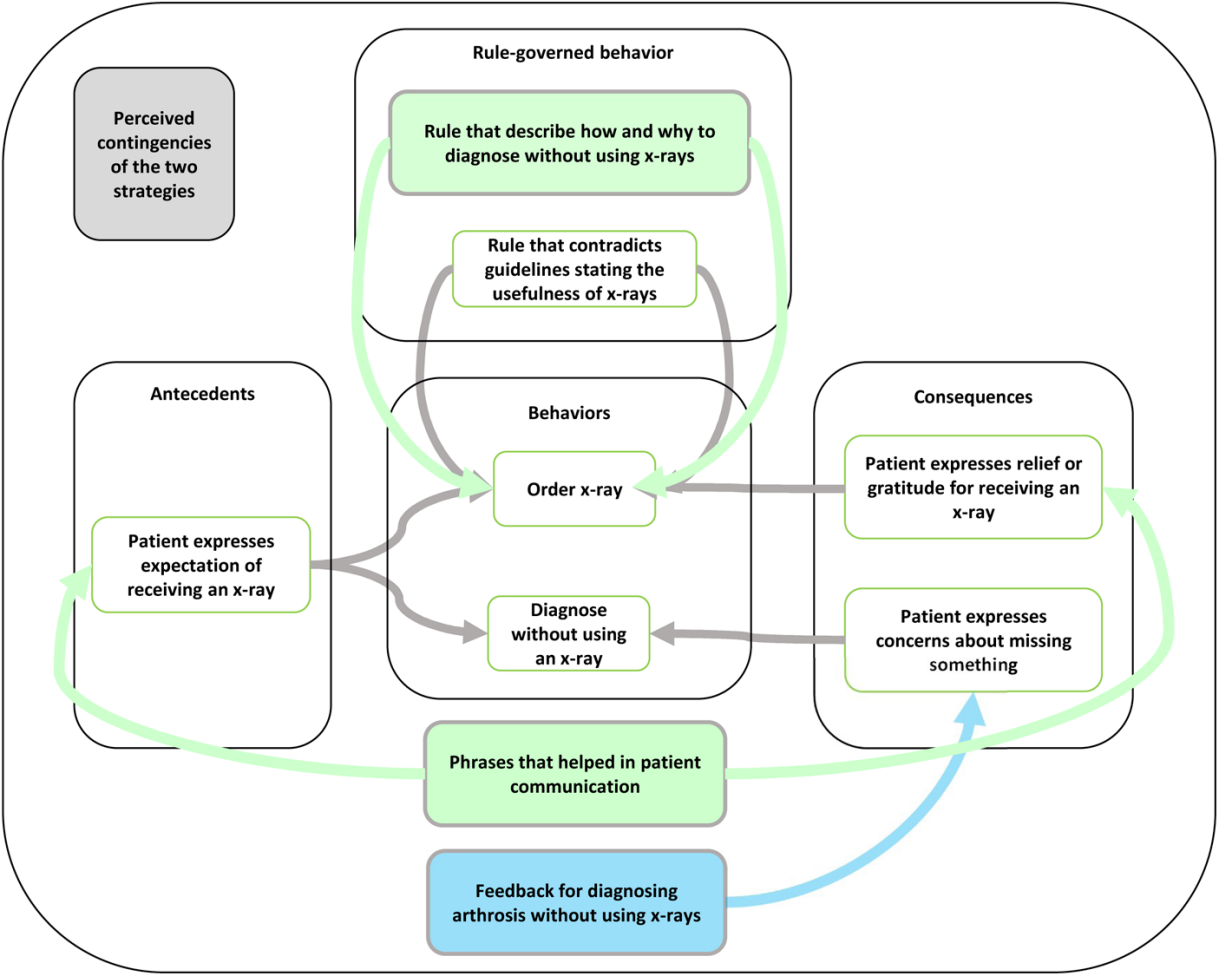
Key principles, concepts, participants’ perceptions of the mechanisms specific to the use of x-rays, and how the strategies targeted those mechanisms. The green boxes show how both strategies influenced the targeted contingencies, and the blue box shows how the feedback meetings influenced the targeted contingencies.

We analyzed contingencies using the concepts three-term contingency and rule-governed behavior, comparing the participants' descriptions to the analysis from before development of the strategies (see Figure 1). The comparisons confirmed the relevance of the contingencies underlying the strategies identified beforehand but also indicated that both strategies influenced other contingencies than expected. The lecture (expected) and the feedback meetings (not expected) changed the self-developed rule of needing to satisfy patients' expectations by enabling the physicians to satisfy the patients' expressed expectations without unnecessary x-rays when diagnosing arthrosis (Figure 4).

“I believe that we discussed this during the meeting, and this is what it is mostly about. How you, in a pedagogical way, respond to the patient's thoughts, concerns, and wishes and then to deliver your assessment of it all (symptoms and the patient's perspective). I believe it is easier to avoid unnecessary use of x-rays if you work patient centered” (IP4).

Both the lecture (not expected) and the feedback meetings (not expected) influenced the three-term contingencies related to patients' reactions to not receiving an x-ray. The participants described how they had started to use new phrases while talking to the patients, which influenced the patients' reaction, leading them to express gratitude for their diagnosis without receiving an x-ray.

“And that is something that I find valuable to convey to the patients also, that in an early stage, there is a risk of us underdiagnosing (arthrosis) if we rely on the results from an x-ray. That is a takeaway message from the lecture” (IP4).

The feedback meetings (expected) also influenced the behavior of diagnosing patients with arthrosis without using an x-ray by adding consequences encouraging this behavior. This was done by receiving feedback and providing a more general form of support from talking to their colleagues about issues related to not using x-rays to diagnose arthrosis.

“Above all, I believe that since we were able to talk amongst ourselves and simply be able to reflect and talk about it. That is what I believe was especially valuable” (IP4).

#### 3.4.2. Feasibility

We found four categories related to the participants' perceptions of the feasibility of the applied behavior analysis approach. The participants described it as feasible because the strategies had the potential to influence their behaviors and the approach could be beneficial for other examples of LVC. They also provided suggestions for how the strategies could be further improved.

Overall, the participants found the design and evaluation process feasible. However, not all clearly remembered participating in the initial participatory process of identifying the LVC practice and factors, indicating that the latter was more important to them. Nevertheless, all participants perceived the choice of LVC as relevant. They stated that they had been aware of x-rays being LVC before the implementation of the strategies but that the strategies had been helpful in reducing their use.

“I believe it is reasonable to try to reduce the use. Since it is possible to diagnose arthrosis clinically, is it reasonable both from a financial perspective and based on our goal to avoid unnecessary examinations in general” (IP3).

They also described the chosen strategies as relevant for targeting their use of unnecessary x-rays and stated that the format for delivering the strategies had been well incorporated into their normal collaborations and routines.

“It's good that it came up at the physicians’ meetings and didn’t go on for too long. But we still have the physicians’ meetings regularly, so it was a good forum to take it there” (IP4).

All participants described other examples of LVC, such as lab tests, cardiology examinations, antibiotics, gastroscopy, and colonoscopy, in which a similar approach could be beneficial, including selecting LVC based on their quality assurance system, inviting someone to provide a short lecture on why it is considered LVC, and measuring and providing feedback on their use.

Some suggestions for improvements were also provided, particularly for the strategies. Two suggestions on how to improve the lectures were proposed. The first was to prepare the participants before the lecture or start with an introduction clarifying the purpose of the lecture on reducing the use of x-rays for arthrosis based on the knowledge that they are not necessary. It was perceived as more implied than explicitly articulated. This was described as an effect of the hectic work situation for physicians and the fact that they often dropped in at meetings without being prepared for what was to be discussed.

Another suggestion was to include some sort of practice of diagnosing patients with arthrosis without using x-rays. The idea was that even if most physicians believe that they are capable of diagnosing arthrosis without x-rays, they could possibly do it differently from each other, and there could be a benefit of practicing to see if there were any differences. This was balanced, however, by the benefit of the lecture being brief based on feasibility.

Even though the free discussions were perceived as helpful, it was also suggested that more structure may be warranted. Two main topics to focus on more specifically were suggested. The first one was related to discussing why one would want to do an x-ray for arthrosis for patients who were not interested in surgery. What could be the perceived benefit of ordering an unnecessary x-ray and to discuss how to handle that in a different way with the group.

The other topic was focused more on the interaction with patients who ask for an x-ray. How to understand their perspectives and based on an understanding of that how to be able to convince them that they do not need an x-ray.

A shortcoming of the feedback received during the feedback meetings was that the data were difficult to interpret. Because few patients presented with arthrosis per month at the center, it was difficult to see a clear trend. They further commented that the feedback was not precise enough to ascertain whether the reduced number of x-rays was an effect of correct or incorrect decisions. Because some x-rays are warranted for pre-surgical consultation, the data was difficult to interpret.

“Perhaps one would have to dive deep into individual cases to check if the results were based on us not using x-rays or not using x-rays for arthrosis” (IP3).

## 4. Discussion

In this study, we used applied behavior analysis to identify two strategies, a lecture and feedback meetings, to address the local contingencies (antecedents, consequences, and rules) maintaining the use of LVC. The results from the evaluation of how each of the three target behaviors changed following the strategies were inconclusive. However, the findings that more patients received the arthrosis diagnosis without an x-ray and more received arthrosis diagnoses than general knee-pain diagnoses after the introduction of the strategies may indicate a behavior change in the expected direction. Such a conclusion is supported by interview data showing that participants perceived an effect in response to both strategies. Qualitative findings showed that the participants described the applied behavior analysis approach as feasible, supported the identified strategies' appropriateness, and suggested additional ways the strategies influenced the contingencies.

The strategies used in this study are consistent with the literature on de-implementation indicating that education and feedback, separate or together, are effective for de-implementation (39–41), and that feedback as a general strategy is effective in changing behaviors (42). Based on the ERIC taxonomy (43), the education and feedback strategies in this study could be sub-classified as including an educational meeting, educational outreach (the invited physiotherapist), development of educational materials (a PowerPoint presentation), and a mandate for change (the presence of the center's manager and medically responsible physician). Such sub-categorization could increase the precision with which a de-implementation effort is described. However, from the perspective of applied behavior analysis, *how* the strategies influence contingencies is more important to describe. One strategy may include several features to maximize the likelihood that the strategy targets the identified contingencies. For example, the aspiration to establish a new rule to govern LVC behaviors was taken into account in the design of the lecture. Features included in the lecture to strengthen the effect as a rule governing behavior was how the PowerPoint presentation was designed, the presence at the lecture by the manager and the medically responsible physician, the inclusion of detailed instructions on how to diagnose arthrosis without using x-rays, that is, a replacement behavior (44), and that the presentation at the lecture was held by an expert i.e., the physiotherapist. Similarly, the discussions held during the feedback meetings aimed to allow for problem solving to influence the three-term contingencies, a strategy that has been shown to be effective in previous studies (45, 46). Therefore, whereas the ERIC taxonomy may provide more details on the available strategies, applied behavior analysis focuses on the strategies' functions—that is, how they are expected to influence behaviors (i.e., mechanisms). This way of designing strategies corresponds well with recent research in applied behavior analysis on how to match an analysis of the target behaviors with relevant strategies offering a way to bridge general knowledge on what strategies influence behaviors and how, with detailed information about a specific context (47).

The single-case data did not consistently point in one direction regarding whether the strategies influenced LVC-related behaviors. Yet, the pattern of change adds a layer to the analysis. The three single-case data together show that after the introduction of the strategies, more patients received the arthrosis diagnosis than x-rays and/or the general knee-pain diagnosis. This may indicate that more patients receive the diagnosis without an x-ray, which aligns with the aspired behavioral change. Interview data also supports this interpretation. Overall, the participants were more positive about the strategies’ perceived effects. Therefore, some discrepancy arose between single-case data and interview data. One reason for this discrepancy could be the turnover rate among the physicians working at the center in combination with the use of center-level data, which meant that behaviors of physicians who were not exposed to the de-implementation strategies were included in the outcome data, which may have reduced the effect. Another explanation could be that interviewees were individuals who experiences the two strategies and therefore may be prone to promoting a positive evaluation simply to justify their time investment or social desirability (48).

Of course, it cannot be ruled out that the strategies simply were not effective. Possible reasons for that could be that the information received from the participants about possible contingencies were not sufficiently comprehensive. Those identified in this study were only a few out of several suggested in the literature (7, 10, 49). Alternative contingencies with a stronger influence on behaviors related to using x-rays could potentially have maintained unnecessary use of LVC despite the two strategies. Another explanation could be that the strategies did not target the maintaining contingencies effectively enough. The feedback intervention, in particular, could have been improved. Feedback is more effective if it is delivered individually, without delays, and if it is delivered from a person who is valued by the recipient of the feedback (42). Thus, access to individual data, more frequent feedback and feedback delivered solely by the medically responsible physician would possibly have improved the effect of the strategies.

One challenge in de-implementation of LVC is that few practices are LVC for all patients (7, 50). For example, ordering an x-ray for patients who are being referred for surgery is still appropriate. This has implications for evaluation of the effectiveness of de-implementation strategies and the design of strategies for de-implementation. From an evaluation perspective, it means that it is unclear whether the results should be interpreted because unnecessary orders of x-rays still occur. Another interpretation could be that more patients were receiving the arthrosis diagnosis than the general knee-pain diagnosis, and the number of ordered x-rays indicates that the strategies contributed to more appropriate ordering of x-rays (i.e., correct decisions). More detailed data and analysis of each patient who received an x-ray would be needed to draw such conclusions but was not available in the current case.

In addition to affecting evaluation, the need for specificity and discrimination between occasions when a practice is of value and when it is not may also influence the design of strategies. The findings from the interviews showed that even though participants confirmed that the two strategies influenced behaviors by influencing the targeted contingencies (lecture influencing rule-governing and feedback meetings influencing the three-term contingencies) and other contingencies (lecture influencing the three-term contingency and feedback influencing rule-governing), they also suggested that the feedback was not specific enough. They wanted feedback on whether the ordered x-rays were based on correct or incorrect decisions, thus pointing to the general challenge in the design of strategies for de-implementation. From a theoretical perspective, the strategies were designed to reinforce and thereby increase one behavior and, as a result, lead to a decrease in another, so-called differential reinforcement (28). Differential reinforcement has been suggested for use in de-implementation (44) but has rarely been used (50, 51). However, the participants emphasized that to target the dilemma of few LVC practices being LVC for all patients, approaches are necessary that improve a behavior's precision so it is only present during the right set of circumstances. In applied behavior analysis, this is called discrimination training (28). Reducing LVC with discrimination training would involve providing feedback on the number of correct decisions (i.e., to order an x-ray or diagnose arthrosis without an x-ray when necessary). The feedback would then improve signal detection (i.e., the ability to identify the correct signal to respond to), thereby increasing the precision with which a strategy is applied (52, 53). A similar argument has been expressed in relation to prevalence data for LVC. Most prevalent data are presented *via* so called indirect or volume measures suggesting that less is always better. However, direct measures or value measures of how many patients who should *not* receive a practice would be a more suitable way of measuring prevalence of LVC (54).

To improve how de-implementation is evaluated and strategies are designed, sufficiently precise data is therefore necessary. In our case and in many other clinical settings, such data may not always be available or would substantially increase the burden of data collection. For example, it may require a person trained in the guidelines reviewing the electronic health journals for all patients receiving an x-ray or one of the two diagnoses to determine how often they were used correctly vs. incorrectly, a task that would be very time consuming, thus making the strategies less feasible. An alternative would be to provide a more intense training with fictive patient cases to deliver precise feedback. A third alternative would be the physicians ordering an x-ray to document whether they were referring the patient for surgery so the data could show correct vs. incorrect decisions. Based on our participants' suggestions for improvement, it may also be sufficient to improve the two strategies used in this study by strengthening their function as rules by more clearly showing how to discriminate between when the practice is valuable and when it is not or by improving the influence on the three-term contingency (more specific problem solving during the feedback meetings).

Implementation science and applied behavior analysis have similar aims, to change socially significant behaviors to create meaningful change. Implementation science has contributed with empirical studies of many different types of strategies available for both implementation and de-implementation purposes. Applied behavior analysis adds to this by using a theory of human behavior that has been applied across settings for decades, providing a way to understand which factors, out of a multitude, that need to be addressed to change behaviors as well as providing a structure for analyzing which strategies could address these factors. Thus, applied behavior analysis may provide a valuable addition to the field of implementation by offering a theoretically guided way of matching strategies to barriers.

### 4.1. Implications for research and practice

The study's results are inconclusive but have some implications for research and practice. The participants found the approach feasible, perceived positive results from the two strategies, and suggested further improvements of the strategies and how they could be used for other examples of LVC. This suggests that using applied behavior analysis to plan and evaluate strategies for de-implementation could be valuable. To improve the approach, knowledge from discrimination training could be used. The approach could also benefit from a continuous improvement approach by being used in several iterations in which feedback from the professionals is used to improve the strategies, which are tested again and improved based on feedback again, making them more precise in their influence on the targeted behaviors [similar to, e.g. (55),]. Similar steps as those taken in this study could be taken in practice to tailor strategies to local contexts and evaluate their effects. “Perfect” data is rarely available in practice but could be good enough to be used for improvements in health care (56).

### 4.2. Methodological considerations

The study has some limitations that need to be recognized when one interprets the findings. This is a small study of one primary care center, which limits our ability to draw firm conclusions and generalize results. However, the combination of quantitative and qualitative data enabled a comprehensive investigation of the process and the two strategies, including their perceived strengths and limitations.

Furthermore, the COVID-19 pandemic may have impacted the results. The general belief at the center was that patients were returning to a more normal level of help seeking during the time of the study. However, if patients had waited to seek help during the pandemic, this may have resulted in increased symptom severity. This could indicate a higher likelihood of patients needing an x-ray for referral to knee surgery. As the patient population gradually returned to normal, a decrease in the number of patients needing x-rays would be natural. We tried to compensate for that by examining a longer period for the data (see APPENDIX 1 in the Supplementary Material) and subjectively evaluating the development of patient visits to the center in general to decide on a reasonable time frame for the baseline data and the study.

Another limitation of the study was the lack of individual-level data. The administrative system did not allow us to extract data on the individual physician level, which may have influenced the results because the number of physicians at the center can influence the number of patients who could be referred for an x-ray. It was also not possible to extract only data regarding the physicians who had participated in the lecture and the feedback meetings, which diluted the strategy's effect. However, the two strategies could also have influenced the entire center even though not everyone participated. The physicians likely discussed the study topic with colleagues and other professions outside of the meeting. The lack of individual-level data also made the feedback component less effective because the feedback never included information on whether each decision had been right or wrong. In theory, a decision not to use an x-ray could have been the wrong decision, and the decision to use an x-ray could have been right. We tried to control for this by also providing feedback on how many patients received the arthrosis diagnosis during the same time frame under the assumption that the more confident the physicians would be in diagnosing patients with arthrosis, the more patients would receive the diagnosis in relation to the number of patients who received an x-ray.

The study also has several strengths. It provides a theoretical approach to de-implementation that makes it possible to analyze influencing factors related to the use of LVC and the mechanism underlying strategies for de-implementation. Our detailed analysis also makes it possible to understand how the same types of strategies can work differently depending on how they manage to influence the targeted contingencies. It also shows that different strategies can work in the same way, by influencing the same contingencies.

## 5. Conclusions

The findings illustrate how applied behavior analysis can be used to analyze contingencies related to the use of LVC and to design strategies for de-implementation. It also shows an effect of the targeted behaviors even though the quantitative results are inconclusive. The conclusion from the qualitative analysis widens the understanding of how different strategies influence existing contingencies related to the use of LVC. The strategies used in this study could be further improved to target the contingencies better by structuring the feedback meetings better and including more precise feedback.

## Data Availability

The raw data supporting the conclusions of this article will be made available by the authors, without undue reservation.
