# Supplementary material for: Strategies to reduce low-value care – An applied behavior analysis using a single-case design
Source: Front Health Serv. 2023 Feb 28;3:1099538. doi: 10.3389/frhs.2023.1099538 (PMC10012739; doi:10.3389/frhs.2023.1099538)

APPENDIX 1. Additional visual presentation of the single case design data.

**Figure 1**

*Extended baseline data for ordered x-rays. Time sequence used in the manuscript is marked by a box. The arrow indicates when the new guidelines were published.*

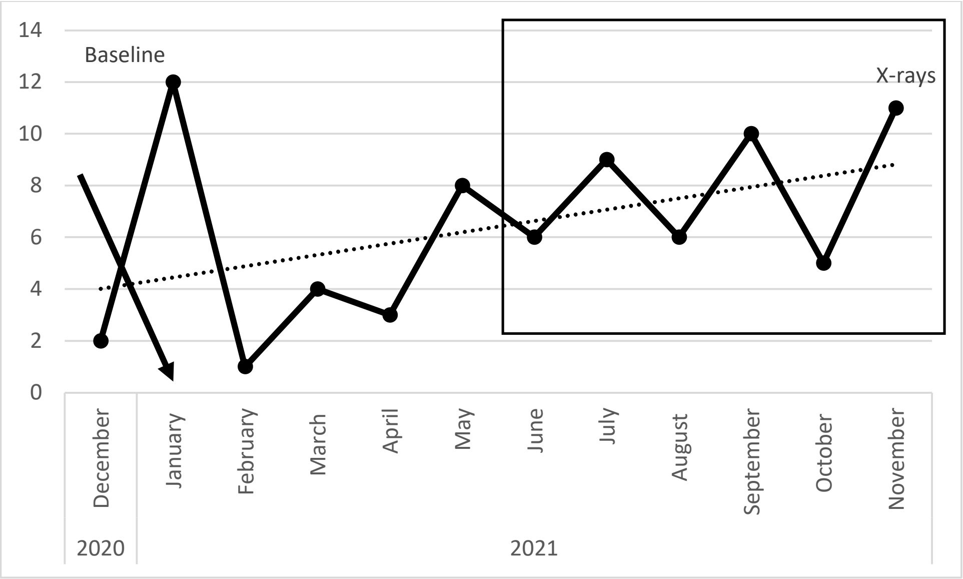


**Figure 2**

*Extended baseline data for number of patients receiving the diagnoses arthrosis. Time sequence used in the manuscript is marked by a box. The arrow indicates when the new guidelines were published.*

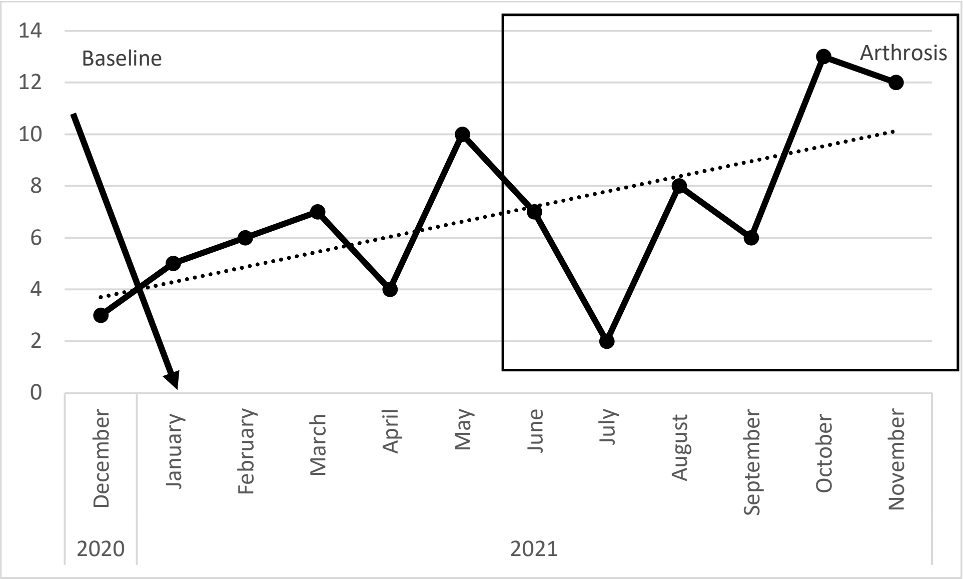

**Figure 3**

*Extended baseline data for number of patients receiving the diagnosis general knee-pain. Time sequence used in the manuscript is marked by a box. The arrow indicates when the new guidelines were published.*

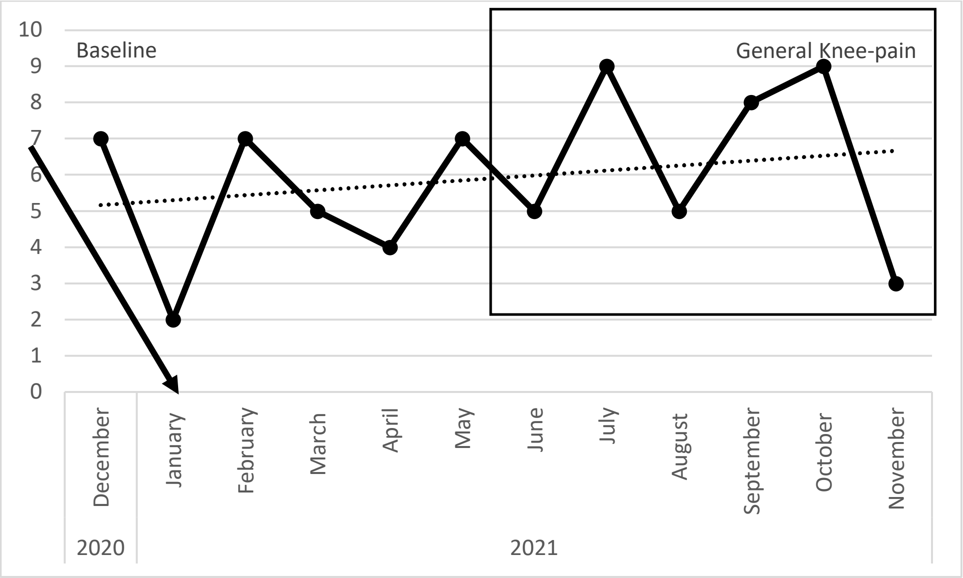

**Figure 4**

*Trendlines for all data and phases.*

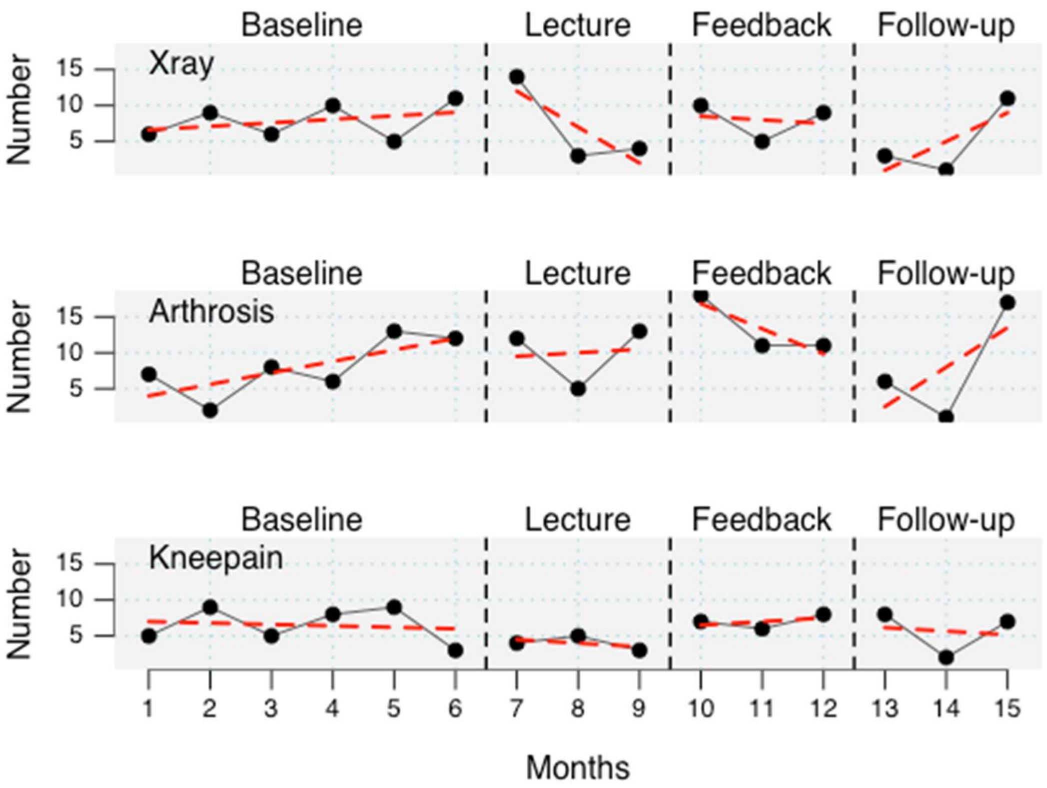

**Figure 5**

*All phases compared to trend for baseline.*

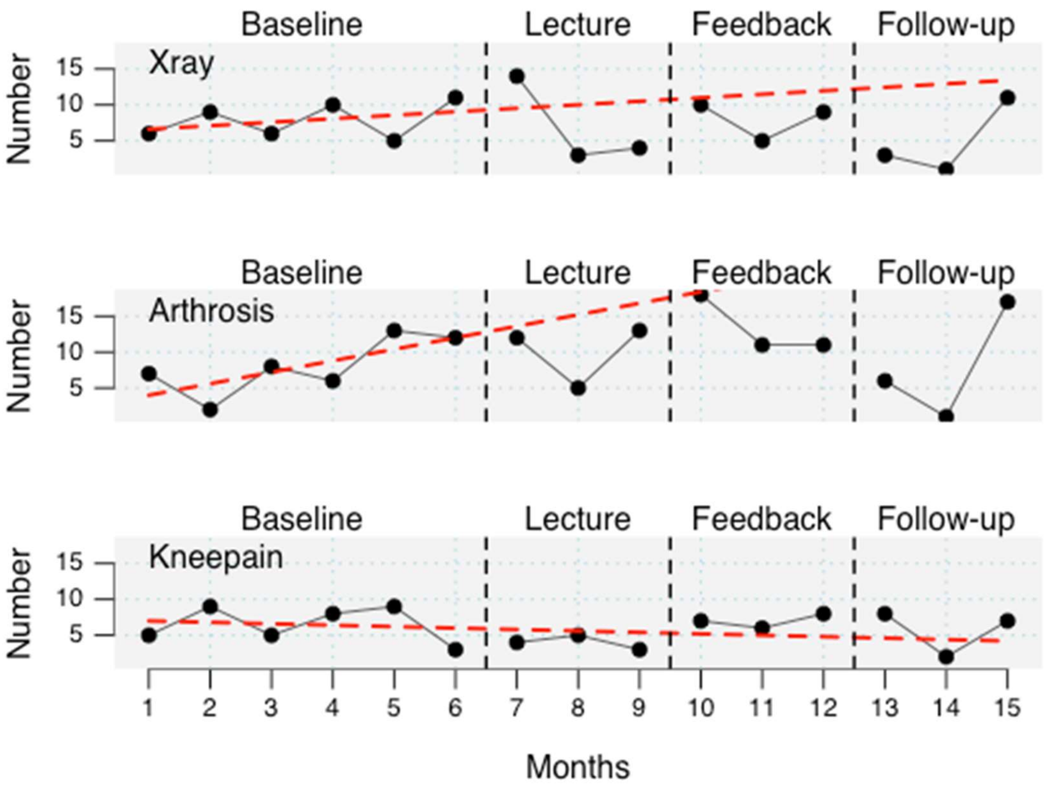

**Figure 6**

*Mean, upper and lower control limit for number of x-rays ordered for all phases.*

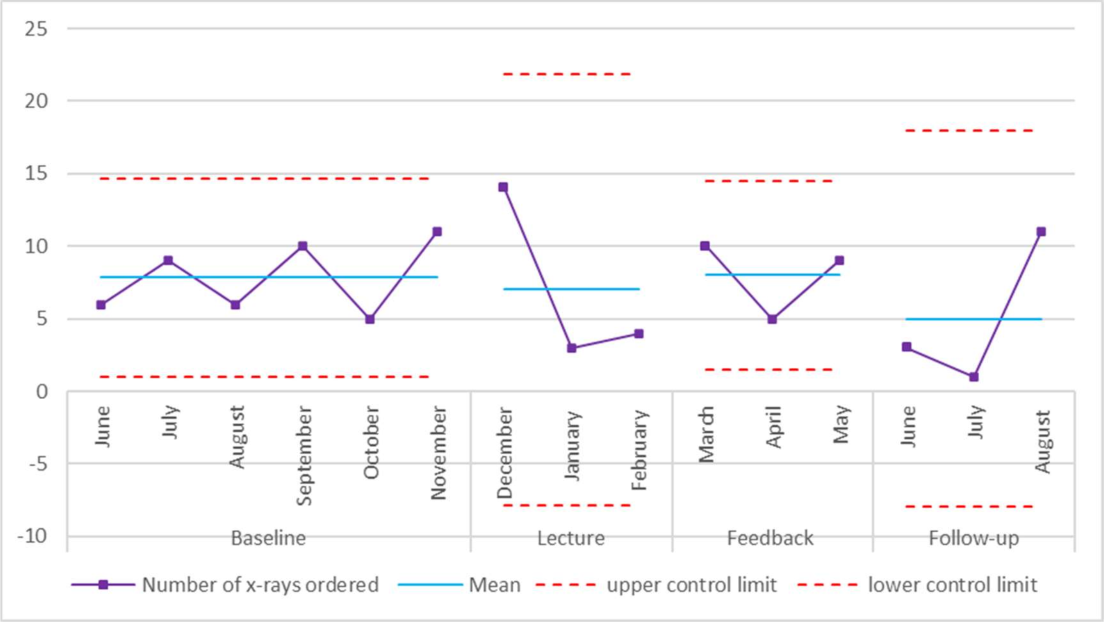


**Figure 7**

*Mean, upper and lower control limit for number of patients diagnosed with arthrosis for all phases.*

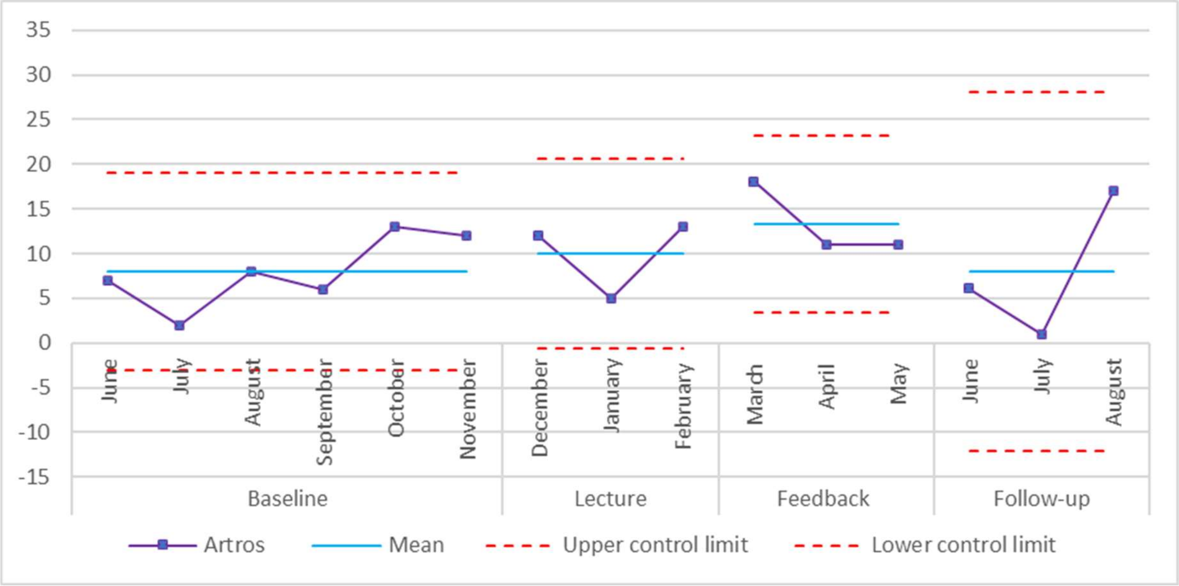

**Figure 8**

*Mean, upper and lower control limit for number of patients diagnosed with general knee pain for all phases.*

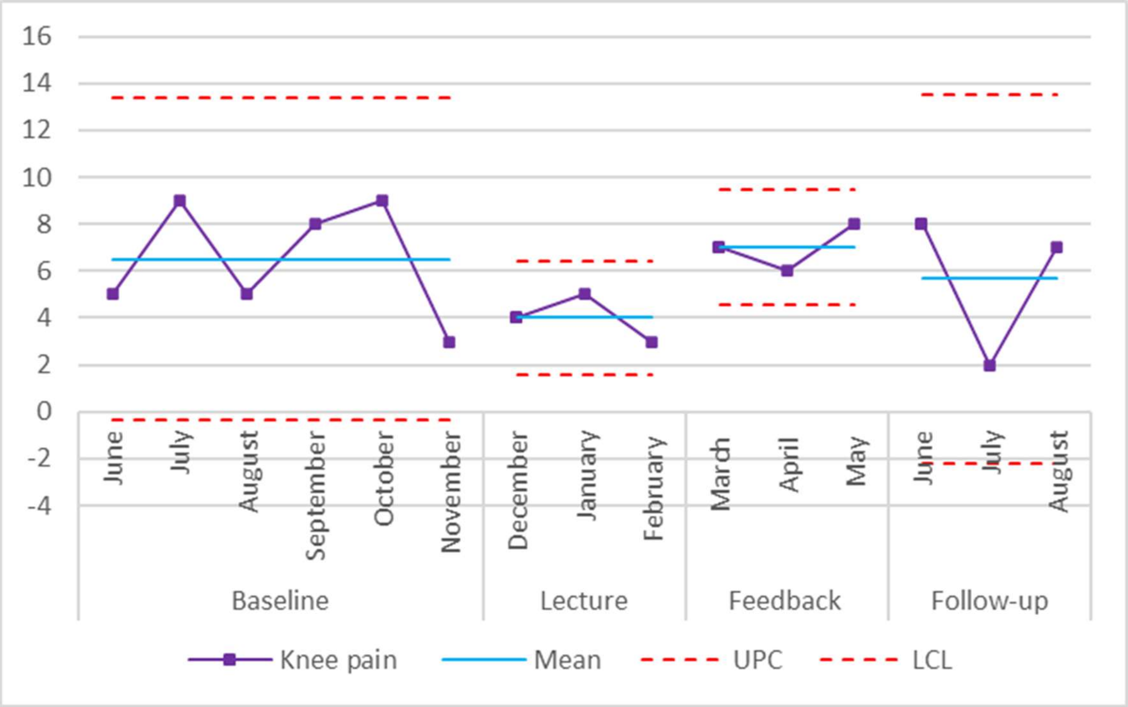

Supplement: Supplementary file 1 [file Presentation1.pdf]
